# Supplementary material for: Time-series modeling of epidemics in complex populations: Detecting changes in incidence volatility over time
Source: PLoS Comput Biol. 2025 Jul 11;21(7):e1012882. doi: 10.1371/journal.pcbi.1012882 (PMC12266409; doi:10.1371/journal.pcbi.1012882)
Supplement: S1 Text — (PDF) [file pcbi.1012882.s001.pdf]

S1 Text. Derivation of the relationship between the dispersion parameter and the mean crowding parameter.

1. Let m^* be mean crowding and m be the mean.
$m^* = m + (\frac{\sigma^2}{m} - 1)$ (by definition of mean crowding)
$\implies m^* - m = \frac{\sigma^2}{m} - 1$

2. Let θ be the dispersion parameter and σ^2 be the variance of the negative binomial distribution.
$\sigma^2 = m + \frac{m^2}{\theta}$ (by definition of negative binomial variance)
$\implies \frac{\sigma^2}{m} = 1 + \frac{m}{\theta}$
$\implies \frac{m}{\theta} = \frac{\sigma^2}{m} - 1$

3. $\frac{m^*}{m} - 1 = \frac{1}{\theta}$ (by equating the left-hand sides of the results in 1 and 2 and dividing by m)
$\implies m^* = m + \frac{m}{\theta}$
