# Supplementary figures and images for: Time-series modeling of epidemics in complex populations: Detecting changes in incidence volatility over time
Source: PLoS Comput Biol. 2025 Jul 11;21(7):e1012882. doi: 10.1371/journal.pcbi.1012882 (PMC12266409; doi:10.1371/journal.pcbi.1012882)

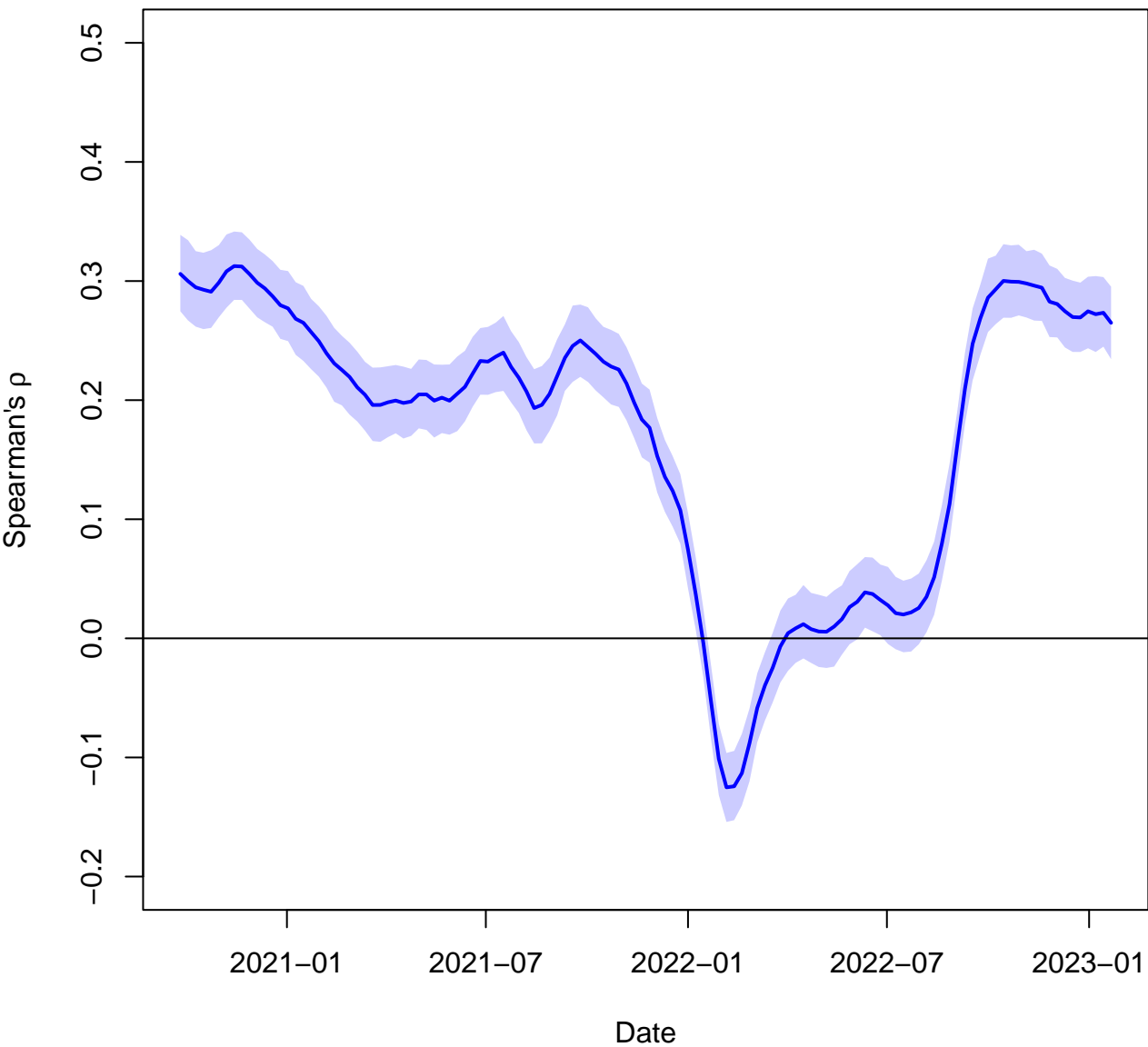

Supplement: S1 Fig — A 32-week sliding window is used to compute each correlation, and 1,000 bootstrap replicates in which county labels are permuted are used to compute the 95% confidence interval. (PDF) [file pcbi.1012882.s002.pdf]
